# Optimizing conversations on treatment management in hereditary angioedema: healthcare professional and patient perspectives on long-term prophylaxis and shared decision-making

**DOI:** 10.1186/s13223-026-01049-7

**Published:** 2026-07-24

**Authors:** Markus Magerl, Emel Aygören-Pürsün, Jens Greve, Andreas Recke, Petra Staubach-Renz, Inmaculada Martinez-Saguer, Lucia Schauf, Diane Langenbacher, Danielle Christmas, Tamara Ray, Sven Pohl

**Affiliations:** 1 Institute of Allergology, Charité-Universitätsmedizin Berlin, Freie Universität Berlin and Humboldt-Universität Zu Berlin, and Immunology and Allergology, Fraunhofer Institute for Translational Medicine and Pharmacology ITMP, Berlin, Germany; 2 Department for Children and Adolescents, University Hospital Frankfurt, Frankfurt, Germany; 3 Department of Otorhinolaryngology, Head and Neck Surgery, Ulm University Medical Center, Ulm, Germany; 4 Department of Dermatology, University of Lübeck, Lübeck, Germany; 5 Department of Dermatology, University Medical Center, Mainz, Germany; 6 HZRM Hämophilie Zentrum Rhein Main, Gartenstraße 134, 60596 Frankfurt/Main, Germany; 7 HAE Vereinigung E.V. [HAE Association], Aldenhoven, Germany; 8 Master Factory for Patient Centric Healthcare GmbH, Neuried, Germany; 9 Research Partnership, London, UK; 10 BioCryst Pharmaceuticals, Inc., Durham, NC USA; 11 BioCryst Pharmaceuticals Deutschland GmbH, Munich, Germany

**Keywords:** Hereditary angioedema, Long-term prophylaxis, Shared decision-making

## Abstract

**Introduction:**

Hereditary angioedema (HAE), characterized by unpredictable attacks of subcutaneous or submucosal edema, can significantly impact patient quality of life (QoL). Despite advances in long-term prophylaxis (LTP), achieving complete control of HAE is challenging, making shared decision-making (SDM) critical for tailored HAE management. This investigation explores the dynamics of healthcare professional (HCP)–patient conversations concerning HAE management, and identifies barriers to SDM, LTP initiation and strategies that may overcome these to optimize patient QoL.

**Methods:**

The investigation was conducted in Germany. HCPs managing patients with HAE participated in 60 min interviews and led simulated patient consultations. 30 min interviews with patients with HAE were also conducted.

**Results:**

Ten HCPs and eight patients with HAE were interviewed. In the simulated consultations, most HCPs recommended LTP based on high attack frequency and substantial impact on QoL. Interviews revealed that HCPs typically initiate discussions on LTP by assessing disease burden, focusing on attack frequency and QoL. These treatment discussions also highlighted the need for improved communication with patients about the LTP treatments that are available to them. However, many HCPs lacked awareness of updated treatment guidelines and faced challenges in reassuring patients about the long-term safety and efficacy of newer LTP options. All patients who were initiated on LTP experienced positive results, including improved QoL and reduced fear of attacks. Those who declined LTP cited low attack frequency on their acute treatment and concerns about burden of treatment and long-term effects. Key barriers to effective SDM included time constraints during routine consultations, absence of clear SDM guidance, and a lack of jargon-free information to foster proactive patient engagement.

**Conclusion:**

This research highlights opportunities to enhance HCP–patient conversations concerning the management of HAE. Inconsistencies between positive patient experiences with LTP and real-world prescription rates emphasize the need for improved SDM practices. Enhancing HCP awareness of patient perspectives, managing time constraints in consultations, and providing unbiased, patient-friendly information may help bridge these gaps and improve communication and ultimately patient QoL. The findings underscore the importance of further research to develop guidelines that prioritize SDM and patient empowerment in HAE management.

**Supplementary Information:**

The online version contains supplementary material available at 10.1186/s13223-026-01049-7.

## Introduction

Hereditary angioedema (HAE) is a rare genetic disorder characterized by severely debilitating attacks of subcutaneous or submucosal edema that can be unpredictable with respect to frequency, severity, and the site of swelling [1]. HAE is associated with significant morbidity as attacks can impair a patient’s ability to perform daily activities, inhibit social activities, and decrease productivity at work or in school [2]. Laryngeal attacks, experienced by over 50% of patients with HAE during their lifetime, pose a life-threatening risk as swelling can lead to asphyxiation and, in some cases, death. The unpredictable nature of HAE highlights the critical importance of effective management of the disorder [2, 3].

Substantial advances in the management of patients with HAE have been made in the past decade. Effective, long-term prophylaxis (LTP) options now offer patients with HAE a level of control over their attacks not previously experienced with traditional acute, on-demand medications, leading to significant improvements in quality of life (QoL) [1, 4]. Consequently, the World Allergy Organization/European Academy of Allergy and Clinical Immunology (WAO/EAACI) guidelines have been updated to reflect the primary goals of treatment in patients with HAE: achieving complete control of the disease and normalizing patients’ lives, a feat currently only attainable with LTP [5]. The approach to patient management has therefore shifted towards designing individualized LTP treatment strategies tailored to optimize patient QoL [6], by considering factors such as disease activity, the ability to control attacks with on-demand therapy, and patient preferences [5].

However, successful LTP treatment requires a high degree of patient compliance, highlighting the importance of healthcare professionals (HCPs) gaining a deeper understanding of patients’ expectations and treatment preferences [5]. In this context, shared decision-making (SDM) for treatment selection emerges as a collaborative approach that can help HCPs better understand patient goals, empowering patients to take control of their disease and ultimately, improve their QoL [2, 6].

In this investigation, we conducted simulated consultations with HCPs responsible for managing patients with HAE, alongside interviews with HCPs and patients. The objectives of the investigation were to gain insights into current conversations between HCPs and patients, identify barriers to the individualization of treatment with SDM and the use of LTP (especially following the updated WAO/EAACI guidelines), and explore strategies to effectively overcome these barriers to optimize patient QoL.

## Methods

### Design

**Fig. 1:**
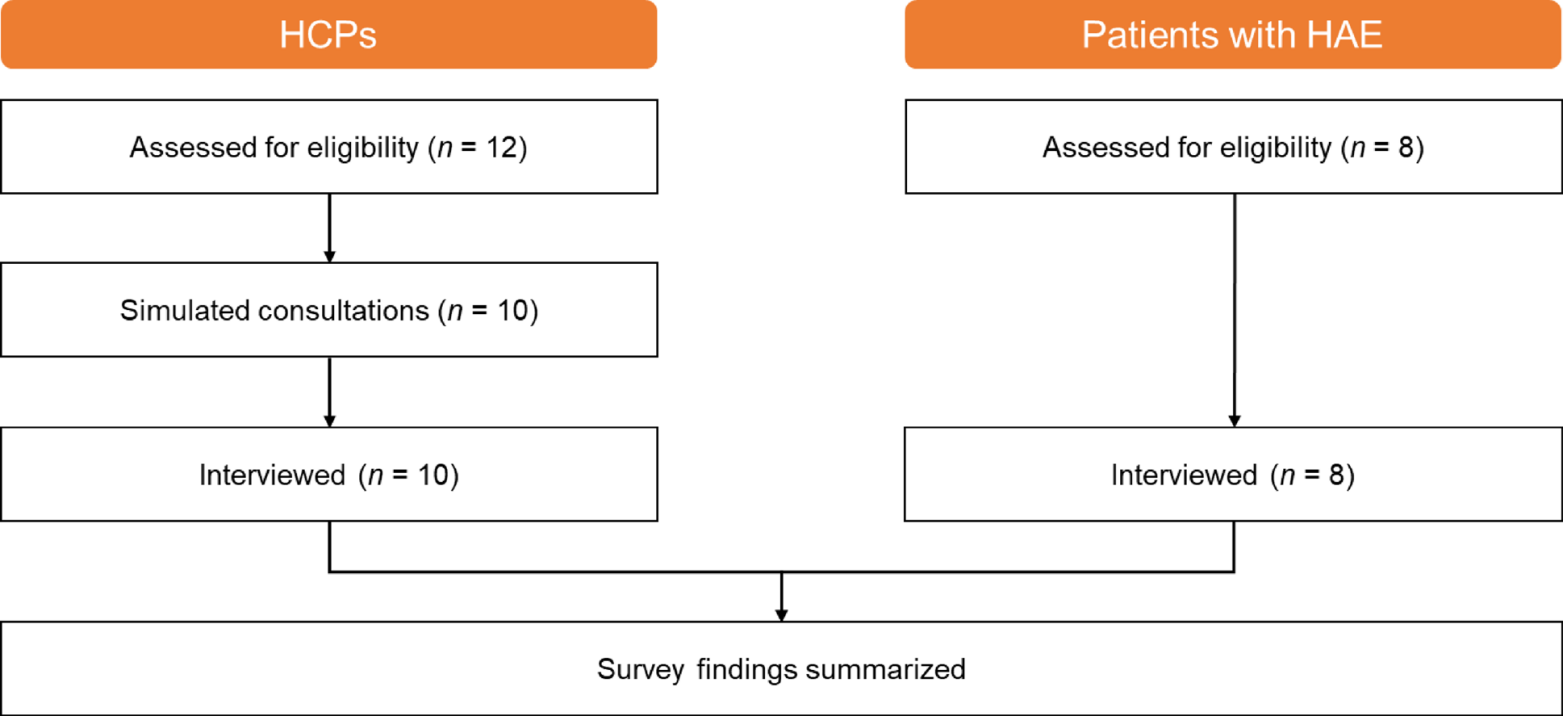
Investigation design HAE, hereditary angioedema; HCP, healthcare professional

Interviews were conducted with patients with HAE and HCPs from 20 to 27 October 2022. HCPs also led two simulated consultations with actors who portrayed patients with HAE. All conversations were conducted in German and subsequently translated into English for analysis (Fig. 1).

All recruitment and research were conducted in accordance with the European General Data Protection Regulation and the European Pharmaceutical Market Research Association code of conduct. Anonymity of HCPs and patients was maintained throughout.

### HCP and patient selection criteria

HCPs and patients who had previously expressed interest in participating in market research studies, surveys, or other activities were primarily recruited from a pre-existing independent database panel. Participating HCPs and patients received honorariums.

Through the panel provider, HCPs (including dermatologists, allergists, Ear, Nose, & Throat specialists, pediatricians, immunologists, and internists) were contacted for their consent to participate in this research if they met all of the following criteria: (i) had been in practice for 3–35 years in their current specialty, (ii) were currently managing at least three patients with Type 1 or 2 HAE, (iii) were personally responsible for making treatment decisions, and (iv) were open to discussing and/or recommending LTP when appropriate.

Patients were contacted through the panel provider for their consent if they met all of the following criteria: (i) were formally diagnosed with Type 1 or Type 2 HAE for at least 12 months, (ii) had been prescribed a treatment for the acute management of HAE, (iii) had previously discussed LTP with an HCP, and (iv) who had been prescribed LTP (currently or in the past) and those who were LTP naïve. Additional patients were recruited through consulting patient advisory groups or patient advocacy groups (PAGs) in Germany who also met the inclusion criteria.

### Simulated HCP consultations

HCPs participated in two simulated consultations and subsequently reflected on their experiences and insights gained during these interactions.

Patient personas (Patient 1 and Patient 2) were developed to illustrate various scenarios (Additional File 1). Patient 1 exemplified a case where the patient was eligible for LTP due to a high frequency of attacks and significant impact on QoL but expressed reluctance to consider it. This scenario aimed to evaluate whether HCPs would engage in discussions about LTP with a patient who met eligibility criteria and whether they would propose LTP as a treatment option.

Patient 2 was designed to represent a scenario where a patient experienced a low attack frequency coupled with moderate QoL impairment but expressed a strong level of interest in starting LTP. The patient had borderline well-controlled angioedema based on Angioedema-QoL [7] and Angioedema Control Test [8] scores. During the first two simulated consultations, the patient reported six attacks in the previous 12 months. This was reduced to four attacks in the previous 12 months in the subsequent eight consultations. This scenario aimed to evaluate whether HCPs would engage in discussions about LTP with a patient who was borderline eligible and if LTP would be recommended as a treatment option.

### Interview structure

Interview questions for both HCPs and patients were designed and refined by the research team (Additional Files 2 and 3).

HCPs participated in 60 min in-depth interviews conducted either in person or via telephone in-depth interview. Interviews focused on discussing their background, current practice, and participating in the simulated consultations.

Patients engaged in 30 min telephone in-depth interviews to discuss their background, reflect upon past conversations on LTP, and explore hypothetical reasons why other patients may choose to accept or decline LTP.

### Interview and consultation data analysis

The interview and consultation data were analyzed using an inductive qualitative content analysis approach, and audio recordings were reviewed in their original German by a native German-speaking qualitative researcher. The content was translated directly into English and organized into structured analytic matrices, enabling systematic comparison across respondents. Translation from German to English occurred concurrently with analysis.

Categories were developed inductively from the data and iteratively refined during analysis. Two additional researchers reviewed the categorized material and collaborated to identify higher-order themes. Interpretations were discussed in analytic meetings, and themes were finalized through consensus. Analysis was conducted manually without qualitative data analysis software.

The number of interviews was predefined based on study feasibility and recruitment considerations. As such, formal assessment of thematic saturation was not an objective of the study; findings should therefore be interpreted as exploratory.

## Results

### Interviewed HCP and patient sample

Twelve HCPs and eight patients were assessed for eligibility. Ten HCPs (Table 1) and eight patients (Table 2) met the inclusion criteria and were invited to participate in the interviews.

**Table 1:** Demographics and characteristics of the interviewed HCPs

Specialty, *n* (%)	
Dermatology	9 (90)
Ear, Nose, & Throat	1 (10)
Length of practice in current role, years	
Mean (range)	16.1 (7–25)
Patients with type 1 or 2 HAE currently managing, mean	
Mean (range)	8.3 (3–20)
Primary practice setting, *n* (%)	
Academic hospital	5 (50)
Medical care center	1 (10)
Office/clinic	3 (30)
Other^a^	1 (10)
Knowledge level of LTP, *n* (%)^b^	
Low	2 (20)
Moderate	1 (10)
High	1 (10)
Very high	6 (60)

HAE, hereditary angioedema; HCP, healthcare professional; LTP, long-term prophylaxis

^a^Time split between academic hospital and office/clinic

^b^Based on perceptions from simulated consultations

**Table 2:** Demographics and characteristics of the interviewed patients

Age, years	
Mean (range)	52 (41–65)
Age at HAE diagnosis, years	
Mean (range)	30.5 (19–40)
Time from diagnosis to discuss LTP with HCP, years	
Mean (range)	15.5 (11–24)
LTP status,* n* (%)	
LTP naïve	4 (50)
Currently taking LTP^a^	4 (50)
Attacks experienced in the last 12 months, *n* (%)	
One	5 (62.5)
Two^b^	2 (25)
Three	1 (12.5)

HAE, hereditary angioedema; HCP, healthcare professional; LTP, long-term prophylaxis

^a^*n* = 2, androgen-receiving patients who were initiated on LTP before HAE-targeted LTP was available to them; *n* = 2, HAE-targeted LTP

^b^One patient reported 1–2 attacks per year

## Research findings

**Fig. 2:**
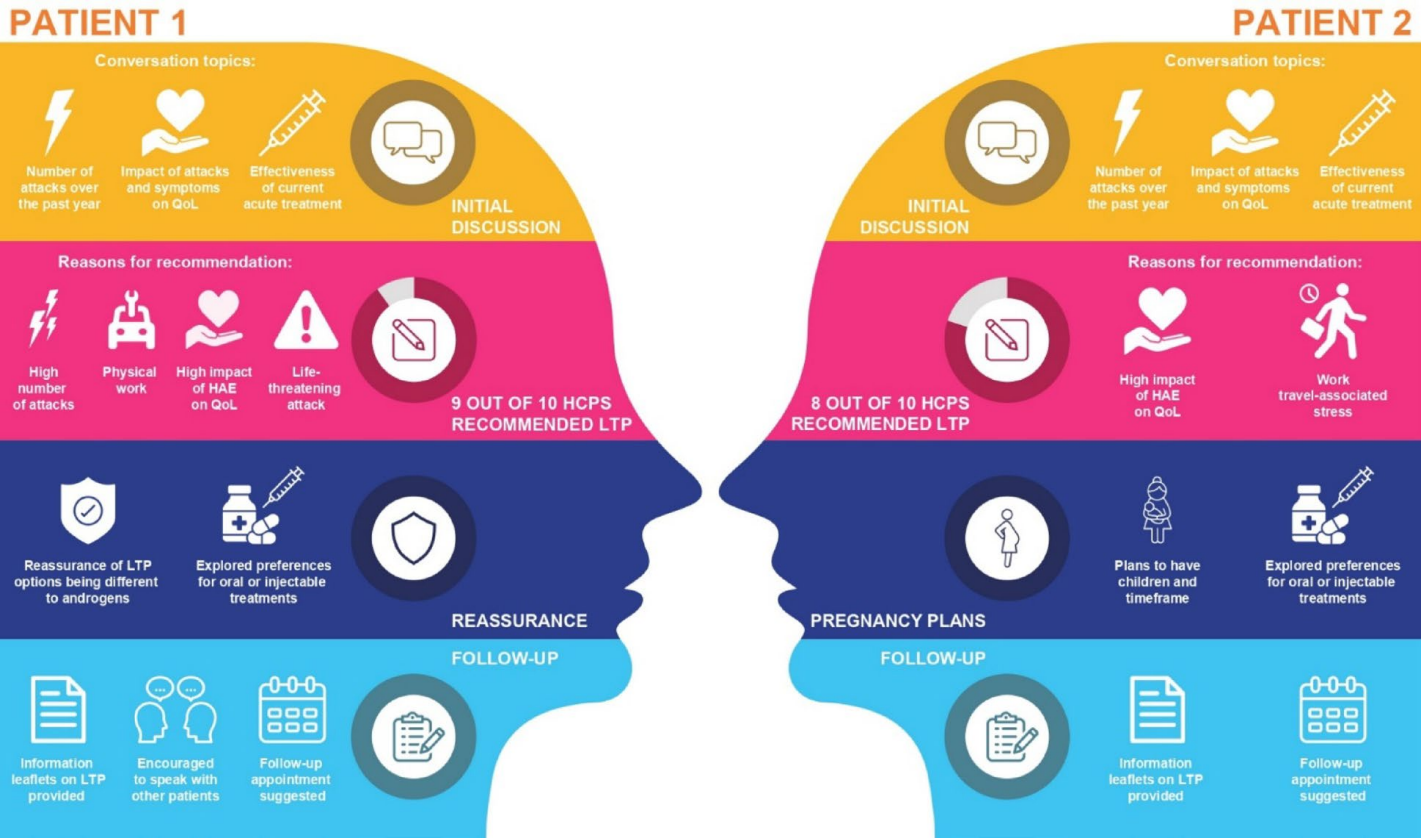
Findings from simulated consultations HAE, hereditary angioedema; HCP, healthcare professional; LTP, long-term prophylaxis; QoL, quality of life

**Fig. 3:**
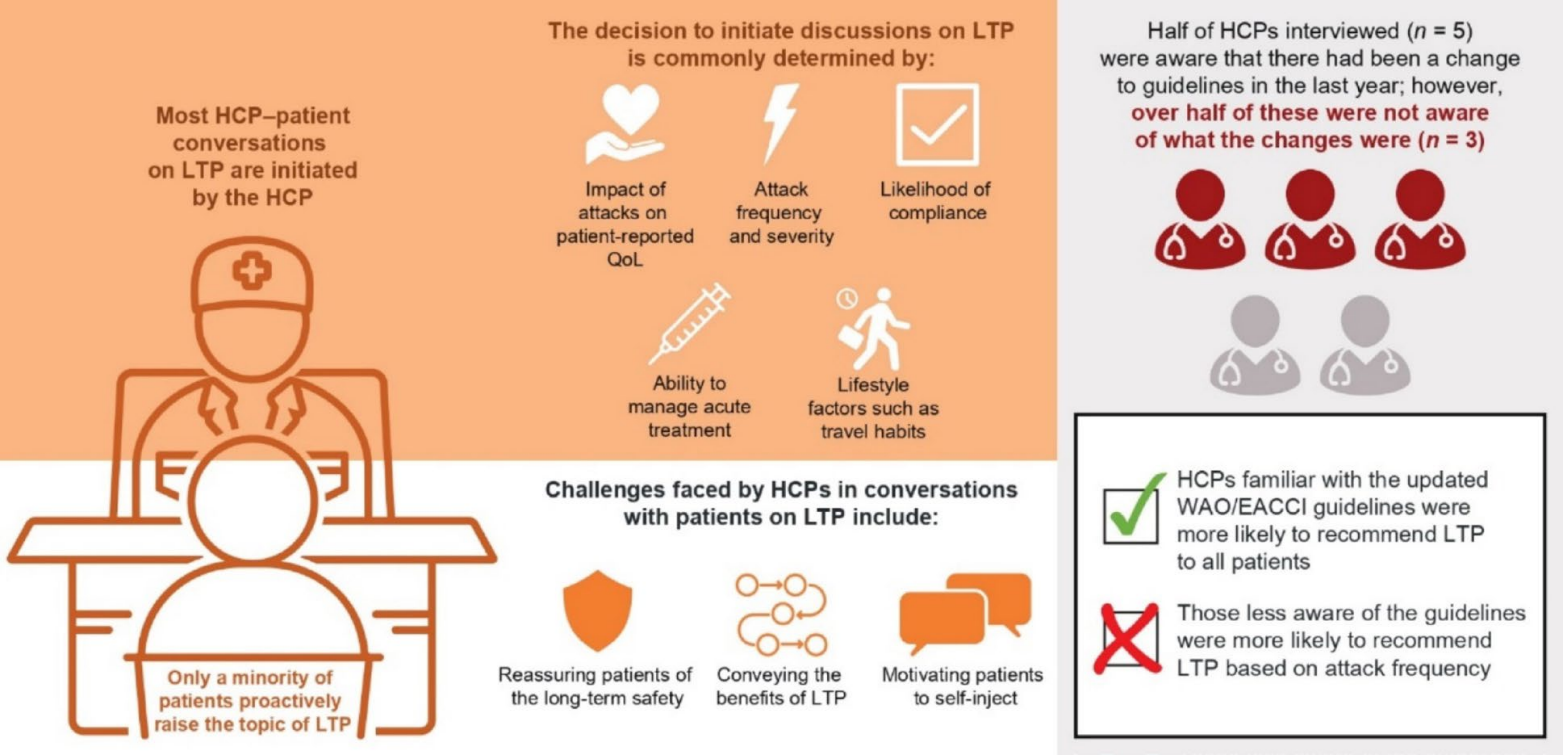
Findings from interviews with HCPs HCP, healthcare professional; LTP, long-term prophylaxis; QoL, quality of life; WAO/EAACI, World Allergy Organization/European Academy of Allergy and Clinical Immunology

**Fig. 4:**
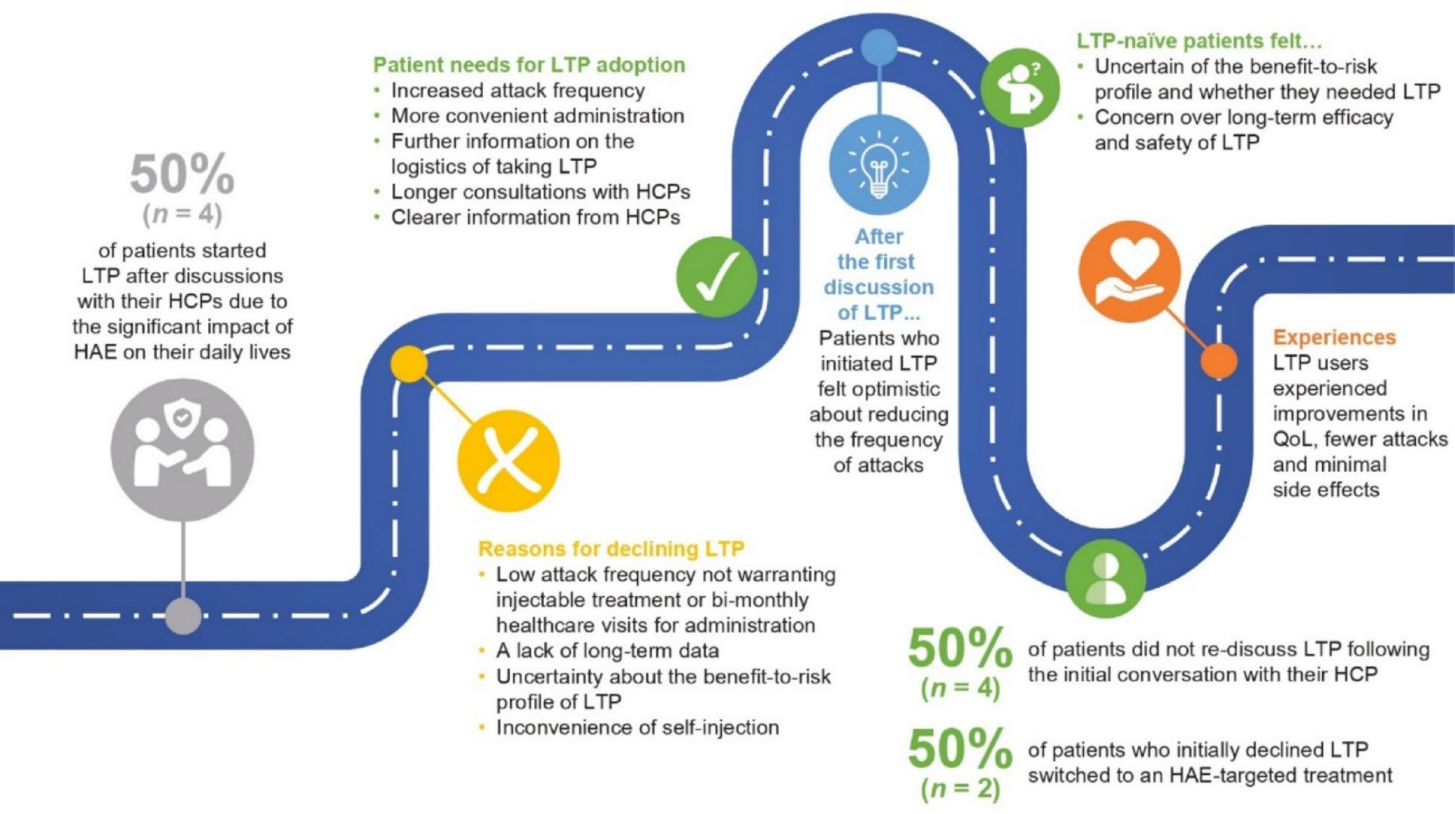
Findings from interviews with patients HAE, hereditary angioedema; HCP, healthcare professional; LTP, long-term prophylaxis; QoL, quality of life

Research findings are presented for the simulated HCP consultations (Fig. 2), interviews with HCPs reflecting on their current clinical practice (Fig. 3), and interviews with patients (Fig. 4).

### Simulated HCP consultations

The decision-making process for LTP recommendations varied based on the characteristics of the simulated patient. In the consultation with Patient 1, HCPs began by assessing how the patient was currently managing their HAE. They enquired about the number of attacks and symptoms experienced by the patient over the past year, the impact on their QoL, the patient’s response time to treat an attack following onset, and the effectiveness of their current acute treatment.

Most HCPs (*n* = 9/10) recommended LTP for Patient 1, highlighting its benefits, such as improved QoL and greater peace of mind. Key factors influencing their recommendation included the high frequency of attacks, the physically demanding nature of the patient’s work, their emotional state, a history of a life-threatening attack, and a high Angioedema-QoL score. However, one HCP chose not to recommend LTP to Patient 1, citing concerns about the patient’s previous liver cell adenoma. For this HCP to reconsider LTP for the patient, they required assurance of a low risk for the progression of the adenoma to malignancy and confirmation that the patient was no longer effectively managing their attacks with acute, on-demand treatment.

Given the reluctance of Patient 1 to start LTP due to prior negative experiences with androgens, most HCPs reassured the patient about the improved efficacy and safety of current LTP options. Some HCPs also explored the patient's preference for oral or injectable treatments and provided information on only one or two specific LTP options. Finally, HCPs provided information leaflets and encouraged the patient to seek further information, as well as to connect with other patients for insight about their experiences with LTP. A follow-up appointment was suggested to allow for further discussion and an opportunity for SDM regarding LTP initiation.

In the consultation with Patient 2, HCPs similarly began by evaluating how the patient was currently managing their HAE, including the number of attacks and symptoms experienced in the past year and the impact on their QoL. The patient’s current acute treatment was also discussed, focusing on its effectiveness and whether any issues had been encountered.

Most HCPs (*n* = 8/10) recommended LTP to Patient 2, highlighting the same benefits as mentioned in the consultation with Patient 1. However, the rationale for recommending LTP to Patient 2 was primarily based on the substantial impact of HAE symptoms on their QoL and the stress associated with work-related travel. Although some HCPs believed that treatment guidelines recommended LTP for patients experiencing ≥ 12 HAE attacks per year, they emphasized the importance of considering the psychological burden on patients, noting that those who have fewer attacks may still experience substantial distress. This focus on QoL was echoed by other HCPs, who emphasized the need to discuss LTP with all patients, irrespective of attack frequency, due to the complexity of each case and their individual needs.

When Patient 2’s profile was adjusted to make them borderline eligible for LTP in the simulated consultation, two of eight HCPs did not recommend LTP. These HCPs argued that the patient was managing their symptoms “superbly well” and believed that a threshold of 12 to 24 attacks per year would be necessary to justify prescribing LTP. Over half of HCPs (*n* = 5/8) also discussed the patient’s plans for pregnancy, highlighting the lack of consensus on the use of LTP and acute treatments during pregnancy and recommended seeking genetic advice from a specialist. Only one or two specific LTP options were explored, and one HCP mentioned insurance coverage considerations due to the patient’s low attack frequency. Finally, HCPs provided information leaflets and suggested scheduling a follow-up appointment for further discussion and SDM.

### Interviews with HCPs reflecting on their current clinical practice

In their interviews, HCPs revealed that they initiate most discussions on LTP with their patients, with only a minority of patients proactively raising the topic. HCPs primarily initiate these discussions based on the perceived impact of HAE attacks on patient-reported QoL, which includes both physical aspects—such as the ability to engage in daily activities, work, hobbies, and travel—and the emotional burden, encompassing the stress, fear, and anxiety associated with attacks. The greater the impact of HAE on patient-reported QoL, the more likely HCPs are to discuss LTP.

The frequency and severity of attacks also influence whether HCPs initiate discussions on LTP. HCPs who are less aware of recent guideline updates often base patients’ eligibility for LTP on the number of attacks experienced per year, with this criterion varying by region, ranging from > 6 attacks to > 12 attacks annually. Discussions about LTP are more likely to be initiated by HCPs if the patient experiences laryngeal or facial attacks, if the attacks occur in a physical region that impacts the ability to work, or if they take a long time to resolve. Other contributing factors include the patient’s ability to manage acute treatment, the likelihood of compliance, and lifestyle factors such as travel habits. These factors encompass how promptly the patient takes their acute treatment, their consistency in carrying medication for emergencies, their likelihood of adhering to regular treatment—especially considering the costs associated with LTP treatments—and the frequency of their travel. A significant challenge cited by HCPs in discussions with patients is reassuring them about the long-term safety of LTP, particularly concerning newer treatments for which long-term data are limited. The complexity of conveying the benefits of LTP and motivating patients to overcome the hurdle of self-injection also presents significant challenges.

Regarding awareness of recent WAO/EAACI guideline changes, half (*n* = 5/10) of the interviewed HCPs were aware of the updates. However, three of these HCPs lacked specific knowledge about the nature of these updates. For those who were aware, the prevailing sentiment was that the updated guidelines aligned with their current management approach, emphasizing the importance of basing LTP recommendations on attack severity and QoL rather than the number of attacks experienced. Conversely, HCPs with limited awareness of the updated guidelines tended to recommend LTP based on attack frequency.

Despite WAO/EAACI guidelines recommending plasma-derived C1-esterase inhibitors, lanadelumab, and berotralstat as first-line LTP treatment options for patients with Type 1 or 2 HAE [5], HCPs predominantly discuss only one or two LTP options in clinical practice. The decision-making process for selecting which LTP treatment to discuss primarily focuses on the potential reduction in attack frequency, with the aim of improving QoL and reducing the burden of disease. Long-term safety and side effects are often considered secondary in this decision. Patient preferences regarding treatment frequency and mode of administration also play a significant role in shaping HCP recommendations. Treatment discussions are primarily initiated and led by HCPs, as patients often seek recommendations from their treating physician. Furthermore, unless patients proactively engage with their HAE management, they often have limited awareness of the available LTP treatment options.

### Interviews with patients

Interviews with patients revealed that half (*n* = 4/8) had started LTP (*n* = 2, androgen-receiving patients who were initiated on LTP before HAE-targeted LTP was available to them; *n* = 2, HAE-targeted LTP) following initial discussions with their HCPs due to the significant impact of HAE on their daily lives. These patients reported feeling optimistic about the potential to reduce attack frequency, with some hopeful that LTP could potentially cure their HAE.

Among patients who were recommended LTP by their HCPs but declined to take it (*n* = 4/8), several factors influenced their decisions. A key reason was the perception of a low attack frequency, which they felt did not justify injectable treatment, especially given that targeted oral LTP options were not available at the time of discussion for some patients. The requirement for bi-monthly healthcare visits for drug administration also contributed to their decisions to decline. Additionally, concerns about the lack of long-term data, the benefit-to-risk profile, and the inconvenience of self-injection also played a role. Uncertainty was particularly pronounced among patients who were managing well with acute, on-demand treatment, and questioned whether the frequency of their attacks warranted starting LTP. Key concerns included treatment cost, potential drug interactions with acute treatments, and the duration of “long-term” treatment. As such, patients sought more information on the long-term efficacy and safety of LTP.

LTP-naïve patients stated that they would consider LTP if they experienced more frequent attacks, such as ≥ 1–2 attacks per quarter instead of 2–3 per year. They expressed a preference for more convenient administration options, such as an oral medication (which was not available to some patients at the time of discussion) or less frequent injections (< 2 per month). Additionally, they sought more information about the logistics of LTP, including where to go for treatment, appointment scheduling, and the expected duration of appointments, including waiting times. Patients also enquired about potential drug–drug interactions, the duration of LTP treatment, and whether with time LTP could cure HAE. Some patients highlighted the need for longer consultations to allow for more thorough discussions about LTP, as short appointment times often leave them feeling rushed and uncertain. They also requested clearer, jargon-free information from their HCPs.

Half of the interviewed patients (*n* = 4/8) did not revisit discussions on LTP after their initial conversation with their HCP. However, patients noted the importance of follow-up discussions with their HCP to reinforce their understanding of LTP as a potential future treatment option. This was especially important as they highlighted that medical jargon often complicated their ability to ask their HCP questions. Among those who revisited discussions on LTP, 50% of patients who initially declined a switch (*n* = 2/4, androgen-receiving patients) chose to switch to an HAE-targeted LTP treatment. Conversely, LTP-naïve patients (*n* = 2/4) again declined LTP during follow-up, citing their perception of having a low attack frequency and expressing a preference for long-term safety data before considering LTP.

LTP users (*n* = 4/8) reported notable improvements in their QoL, experiencing fewer attacks and minimal side effects. Patients highlighted how LTP transformed their lives, allowing them to engage in activities they had previously enjoyed before their HAE attacks started, such as working, pursuing hobbies, caring for family, and traveling. They expressed relief at no longer living in constant fear of attacks and HAE no longer dominating their lives. One patient preferred the convenience of oral LTP, which was available to them at the time, over injectable options, while another patient preferred subcutaneous injections to intravenous administration for ease of use. Overall, users reported fewer attacks after starting LTP, with some even claiming to be attack free, although this may not be directly attributable to treatment.

## Discussion

This investigation provides insights into LTP for HAE from both the HCP and patient perspective. Simulated consultations showed that HCPs were inclined to recommend LTP for patients with high attack frequencies, as well as for those with significant QoL impairments, irrespective of attack frequency. Interviews with HCPs regarding their current practice revealed that they primarily initiate discussions on LTP with patients, focusing on perceived disease burden and attack severity. However, some HCPs showed limited awareness of treatment guideline updates that promote a holistic approach to disease management. Patients expressed varying levels of readiness to consider LTP based on their personal experiences with HAE. Those who started LTP reported improved QoL and reduced anxiety about attacks, while those who declined cited concerns about treatment burden and the sufficiency of their current management. These findings highlight the necessity for more proactive and informed discussions between HCPs and patients regarding LTP, emphasizing SDM and addressing patient concerns about treatment options and administration.

### HCP perspectives on LTP initiation and guidelines

The recommendation of LTP by most HCPs to both patients in the simulated consultations aligns with the known efficacy of LTP in preventing attacks [1, 4, 5] and is consistent with the positive experiences cited by LTP users in this research and the existing literature [9–12]. However, initiating these discussions is a complex process for HCPs, requiring a delicate balance between assessing a patient’s burden of disease and navigating the challenges associated with the chosen treatment approach. The patient personas from the simulated consultations exemplify the real-world complexities faced by individuals living with HAE, offering valuable insights into their decision-making processes and the factors that influence their treatment choices.

It is important for HCPs to understand that the full burden of HAE extends beyond the individual patient to include their families and support networks. This is particularly significant given that familial caregivers often play an active role in supporting those with HAE, and many may themselves be diagnosed with HAE due to its hereditary nature [13, 14]. Understanding these dynamics can enhance HCP engagement in more meaningful discussions about LTP, ultimately leading to more tailored and effective management strategies that consider both the patient’s needs and those of their broader support systems.

The updated WAO/EAACI guidelines reflect a paradigm shift from an attack-specific approach to a more comprehensive and holistic definition of disease burden. These guidelines advocate for treatment goals focused on achieving complete control of the disease and normalization of patients’ lives, which can only currently be achieved with LTP treatment [5]. However, the limited awareness of these guideline updates among HCPs interviewed in this investigation highlights the need for improved educational efforts and communication strategies. Additionally, refining the guidelines to clearly define what constitutes an HAE attack may enhance the understanding of disease burden for both HCPs and patients.

Our research indicates that one of the greatest challenges for HCPs in conversations with patients is reassuring them about the long-term efficacy and safety of LTP, as patients want this information before considering starting treatment. Open-label extension trials have confirmed the long-term efficacy and safety of new LTP treatments indicated for HAE since the completion of this research [15, 16]. It is essential for HCPs to stay up to date with the latest research findings so that they can accurately communicate this to patients, emphasizing that, although LTP treatment has demonstrated a favorable safety and efficacy profile in terms of reducing attacks, unpredictable episodes may still occur. Such nuanced communication may help patients understand that while the dynamics of managing HAE may change when starting LTP, the goal remains to empower them to navigate their condition effectively. By acknowledging patient concerns and providing accurate information, HCPs can foster a collaborative dialogue that supports informed decision-making and SDM, ultimately enhancing the well-being of individuals with HAE.

### Patient experiences and readiness to consider LTP

This research also highlights the reluctance of some patients to initiate LTP due to concerns about treatment burden. A survey of adults diagnosed with HAE in the United States found that more than half of respondents found preventative treatment to be burdensome, and 65% of patients prescribed HAE prophylaxis reported feeling a lack of control over their attacks compared with 44% of those who were not prescribed HAE prophylaxis [17]. While self-administration of HAE treatments can provide patients with a sense of freedom and control over their unpredictable condition, not everyone may prefer or be able to self-administer, and some may be unable to access the support needed from their HCPs or family during an attack [17, 18]. However, other interviews with LTP users have shown that self-administration fosters feelings of control and independence [11], whereas reliance on on-demand or emergency treatment for unpredictable HAE attacks can significantly impair patient QoL [19]. Our findings support this, showing that patients who were initiated on LTP felt optimistic about their future and reported improved QoL and reduced anxiety surrounding attacks. This highlights the crucial role HCPs play in addressing the concerns about starting LTP and the need to optimize conversations around treatment management in HAE. Further research is imperative to effectively define the burden of LTP as the treatment landscape evolves.

Patients’ uncertainties and concerns expressed in this research may drive attitudes of reluctance towards initiating LTP, particularly among individuals who have accepted their HAE diagnosis and have established effective ways to manage their disease in their daily routine. This reluctance is compounded by the historically limited therapeutic options for patients with HAE, such as oral androgens, injections, or infusions, which can present administration challenges and side effects for patients [17]. Consequently, the prospect of introducing LTP into their treatment plan may evoke fears of losing control over their disease and may be met with resistance against adopting a new regimen. While the reasons for hesitancy towards LTP may vary by patient and circumstance [17], patients may need reassurance when transitioning to new treatment regimens. As patients often place considerable trust in their HCPs, which can improve treatment adherence and yield positive patient outcomes as observed in other chronic diseases [20], addressing this need for reassurance is essential to facilitate open communication and partnership between patients and HCPs.

### Barriers to effective SDM in clinical practice

Despite the recognized importance of SDM, this research identified several barriers to the effective use of SDM in real-world practice. For instance, half of patients did not revisit discussions on LTP following the initial conversation with their HCP. This suggests a gap in ongoing patient–HCP communication regarding the consideration and decision-making process related to LTP. Although WAO/EAACI guidelines recommend that patients with HAE should be evaluated for LTP at every visit [5], it is important for patients to be prepared, motivated, and engaged to participate in the SDM process [21], and for HCPs to feel motivated by clear indications that their patients will benefit from revisiting the discussion on LTP. This underlines the importance of SDM, where HCPs can engage in collaborative, patient-centered conversations, empowering patients to decide whether LTP may be a beneficial treatment for their specific case.

Similarly, the LTP treatment options discussed with the patient may be influenced by the HCP’s personal experience and familiarity with specific treatments. While the guidelines state that the choice of LTP should be made through SDM [5], the implementation of SDM in clinical practice is not clearly defined. As a result, some HCPs may limit their discussions to only one or two LTP treatment options, as was observed in this investigation. However, it is important to recognize that some patients may be hesitant to adopt the SDM approach to HAE management and may prefer or expect the traditional, paternalistic model of care [21]. Therefore, HCPs should be encouraged to implement SDM, while also being mindful of the patient’s willingness to engage in the process.

In this research, patients expressed a need for longer consultations to thoroughly discuss LTP and its logistics. These findings align with a cross-sectional study that reported that over 86% of patients with a dermatologic condition, along with their caregivers, valued not feeling rushed during appointments [22]. As SDM requires more time than average consultations in regular care [22, 23], HCPs may need to allocate more time for visits, spread the decision-making process over multiple appointments, and provide patients with information on treatment options before they engage in SDM discussions [6, 21, 24]. However, without a structured communication plan, insufficient time during consultations can hinder the effectiveness of SDM, ultimately making it harder for patients to make informed decisions about their treatment. By addressing these gaps and ensuring more time for patient–HCP interactions, SDM may be more effective [24, 25].

Patients also expressed a desire for clear, jargon-free information in interviews. The success of SDM is intertwined with the level of patient empowerment [21, 24]. SDM can be particularly challenging when patients have limited knowledge of HAE [6, 21, 24], highlighting the importance of ensuring patients are well informed to actively participate in the decision-making process [24]. However, balancing the amount and type of information HCPs provide to patients can be challenging. HCPs must filter information to avoid overwhelming patients, but in doing so, they may unintentionally introduce an informational bias that skews the decision-making process, potentially leading to a more paternalistic approach. Therefore, providing accurate, unbiased, and patient-friendly information about treatment options is essential for effective SDM, though remains a challenge [6]. Creating a structured, supportive environment for SDM discussions is essential, enabling patients to feel empowered and well informed to actively participate in decisions regarding their HAE management.

### Towards a patient-centric SDM approach

To enhance SDM in the management of HAE, future research should focus on developing guidance for HCPs on the questions to ask patients about their daily lives. This may help to build closer relationships between HCPs and patients, which is integral to facilitating effective discussions. A patient-centered approach, guided by the patient’s perspective, is essential to identify their unique needs, preferences, and treatment goals. Facilitating connections with PAGs could also play a key role by providing additional resources and support, helping patients feel more informed and confident in their treatment decisions. Providing translated versions of the WAO/EAACI guidelines, along with concise, patient-friendly summaries endorsed by PAGs, scientific associations, and pharmaceutical companies may further enhance patient understanding. These resources would aim to empower patients to engage in meaningful discussions with their HCPs about the best management options for their HAE.

To streamline the SDM process, future research could explore the use of SMART (specific, measurable, achievable, relevant, and time-bound) questions, which could guide HCPs in discussing concerns, expectations, and treatment goals with patients in a structured, actionable way. Additionally, future iterations of the WAO/EAACI guidelines would benefit from incorporating specific recommendations on the HAE-specific requirements for effective SDM in practice, emphasizing the importance of recognizing patients as experts in their own experiences of HAE, and further promoting a patient-centered approach to treatment decisions.

### Analytical independence and bias mitigation

Given the industry funding of this study, steps were taken to maintain analytical independence and mitigate bias. Qualitative data analysis, including listening to audio recordings, data extraction, category development, theme identification, and the initial interpretation of findings was conducted exclusively by Research Partnership, an independent research agency, without involvement from BioCryst or the key opinion leader authors involved in manuscript development. Analysis was performed by more than one researcher, with themes reviewed and agreed collaboratively as part of a discussion-based consensus, with interpretations grounded in participant interview data prior to manuscript drafting. While these measures were implemented to enhance rigor, the potential for interpretive bias inherent to industry-funded research should be considered when interpreting the findings.

### Limitations

This research has some limitations. As all participants in the research were residents of Germany, findings may not be directly applicable to other countries or healthcare systems. Additionally, despite efforts to eliminate leading questions, variations in how respondents interpreted interview questions could have introduced subjectivity. The simulated consultations format, while useful for capturing HCP decision-making, may have elicited atypical prescribing behavior, and its controlled nature may not fully replicate the dynamics of real-world clinical practice. Furthermore, the HCPs who participated in the interviews had a heterogeneous level of experience in treating patients with HAE. It is unclear how the findings might differ had more HCPs from larger, specialized centers (e.g., those treating > 50 patients with HAE) been included. These limitations emphasize the need for cautious interpretation and limit the broader applicability of the results.

## Conclusions

This research highlights the need to optimize conversations between HCPs and patients on LTP for the management of HAE through SDM. Despite patients’ positive experiences with LTP, there remains a gap in the number of patients who start, discuss, or are recommended LTP by their HCPs. To facilitate effective SDM, it is imperative to enhance HCP understanding of both the treatment burden and disease burden, for patients, caregivers, and families. Managing time constraints in routine consultations, ensuring access to reliable and patient-friendly information on LTP, and providing HCPs with clear guidance on how to implement a patient-centered approach to SDM in clinical practice are key components required for its successful implementation in clinical practice.

## Supplementary Information

Below is the link to the electronic supplementary material.

Supplementary Material 1

Supplementary Material 2

Supplementary Material 3

## Data Availability

The datasets used and/or analyzed during the current study are available from the corresponding author on reasonable request.

## Abbreviations

***HAE***: Hereditary angioedema
***HCP***: Healthcare professional
***LTP***: Long-term prophylaxis
***PAG***: Patient advocacy group
***QoL***: Quality of life
***SDM***: Shared decision-making
***WAO/EACCI***: World Allergy Organization/European Academy of Allergy and ClinicalImmunology
