# Supplementary material for: Optimizing conversations on treatment management in hereditary angioedema: healthcare professional and patient perspectives on long-term prophylaxis and shared decision-making
Source: Allergy Asthma Clin Immunol. 2026 Jul 24;22:44. doi: 10.1186/s13223-026-01049-7 (PMC13401321; doi:10.1186/s13223-026-01049-7)
Supplement: Supplementary file 1 — Supplementary Material 1 [file 13223_2026_1049_MOESM1_ESM.docx]

**Additional File 2.** HCP discussion guide

Introduction (2 minutes)

***Objective: introduction, AE reporting & compliance***

START RECORDING

Good morning / afternoon, thank you for agreeing to take part in this interview. Before we begin, I would like to remind you of a few things that will have already been communicated to you prior to today.

We are conducting this research on behalf of a pharmaceutical company in order to understand more about the management of hereditary angioedema (HAE). The interview will last approximately 60 minutes, and you will receive an incentive as a token of appreciation for your time and contribution to the project.

As part of the interview, we would like you to have a consultation with two different HAE patients. These patients will be played by professional actors, and further information about this exercise will be provided during the interview.

This interview is for market research purposes only – it is in no way intended to be promotional.

There are no right or wrong answers, we are just interested in your thoughts and opinions, and anything you do say will be kept confidential. You have the right to withhold information as you see fit or to withdraw from the interview at any time.

We will be audio recording the interview today, and there are some interested colleagues and clients watching / listening into this interview; however, please be assured that your identity will remain anonymous.

We would prefer not to reveal the name of the sponsoring pharmaceutical company until the end of the interview, just in case knowing this biases any responses.

**Adverse events**

We are required to pass on to our client details of adverse events/product complaints pertaining to their products that are mentioned during the interview. If this happens, we will need to collect details and report the event, even if you have already done so.

Section 1: HCP background (3 minutes)

***Objective: understand HCP background, current HAE caseload & treatments prescribed (spontaneously)***

To begin with, please can you briefly tell me a bit about yourself and the type of setting that you work in (without disclosing your name, or the practice / clinic name):

- Specialty?
- Practice setting e.g. office/ hospital, public/ private?

How many patients do you currently manage, across any condition?

- How many of the patients that you currently manage have been diagnosed with hereditary angioedema (HAE)?
  - How many of your HAE patients have Type 1 or Type 2 HAE?

For the remainder of today’s discussion, when we talk about ‘HAE patients’, please think about your **Type 1 and Type 2 HAE patients only**.

What treatments do you currently prescribe to your HAE patients to help manage their HAE specifically? **Allow for spontaneous response – do not probe / prompt.**

Section 2: simulated HCP–patient consultation (35 minutes)

***Objective: to watch the HCP have two ‘patient consultations’ in order to observe the treatment options discussed, whether long-term prophylaxis (LTP) is brought up as a topic of conversation (and specific LTPs discussed), and what triggers LTP conversations***

As mentioned, we would like you to have a standard consultation (as you would do in practice) with 2 different HAE patients who will be played by performance practitioners (actors specifically trained to understand and portray patients with HAE).

The ‘patients’ will see you for their regular 3-monthly consultation one after the other. You will have approximately 15 minutes with each patient, and we will provide you with a record form for each patient as background information.

We will ask you to treat these patients as you would during any routine consultation and will then discuss the consultations with you when they have finished.

**Moderator: bring in PATIENT 1, and provide HCP with the associated PRF, then leave the room. After 15 minutes, repeat the above exercise with PATIENT 2.**

**Note: in section 3, we will be referring to the content of the consultations, so please take notes in the below box for reference about what the HCP does / doesn’t bring up in the consultations, with particular focus on whether LTP is brought up, why, what treatments are discussed, and what is discussed.**

**PATIENT 1 (~15-minute consultation)**

**PATIENT 2 (~15-minute consultation)**

Section 3: reflection on simulated patient consultations (10 minutes)

***Objective: to reflect on the mock patient consultations had with focus on understanding why LTP was / wasn’t discussed, what triggered discussion of LTP, and rationale for the treatment options that the HCP discussed***

Thank you for completing the patient consultations. I’d now like to spend approximately 10 minutes reflecting on the consultations that you just had.

Let’s discuss the consultation with PATIENT 1 first.

**If LTP WAS NOT discussed:**

- How did you find the consultation with this patient?
- To what extent, if at all, is this patient representative of the types of patient that you see in your practice? Why do you say that?
- I noticed that you didn’t discuss LTP as a treatment option for this patient. Was this something that you considered discussing with them? Why / Why not?
- What were the main reasons why you didn’t discuss LTP with this patient at this time?
  - What would this patient have to disclose to you, or what would trigger you to initiate conversations about LTP with this patient at this time? Why do you say that?

**Allow for spontaneous response and then probe on:**

- - - Number of attacks per year? How many would they need to have?
    - Severity / location of attacks? How severe would they need to be? What location would they need to be in?
    - Patient QOL? What level of impact would it need to be having?
- **PLACEHOLDER FOR ANY QUESTIONS IDENTIFIED WHILST VIEWING CONSULTATION**

**If LTP WAS discussed:**

- How did you find the consultation with this patient?
- To what extent, if at all, is this patient representative of the types of patient that you see in your practice? Why do you say that?
- **If HCP initiated discussion about LTP:** I noticed that you brought up LTP as a topic of conversation during the consultation – why was that / what were the main triggers that led you to discuss LTP with this patient? Why?
- **If patient initiated discussion about LTP**: I noticed that you discussed LTP during the consultation when the patient brought up the topic -
  - What did you think about the patient initiating this topic of conversation? Why?
  - How did you feel about that patient initiating this topic of conversation? Why?
  - If the patient hadn’t raised the topic of LTP, would you have brought it up as part of the discussion with them during this consultation? Why / why not?
- I’d like to understand a bit more about your reasons for discussing specific LTP treatments with this patient
  *
  - I noticed that you discussed [**INSERT LTP TREATMENT**] with this patient as a potential treatment option –
    - **If HCP initiated discussion about the treatment**: why was that / what were the main triggers that led you to discuss this treatment with this patient? Why?
      **Allow for spontaneous response and then probe on:**
    - To what extent, if at all, did you take into account the following factors when discussing this treatment option with this patient –
      - Its mode of administration? Why / why not?
      - The frequency of administration? Why / why not?
      - The long-term safety of the treatment? Why / why not?
      - The tolerability of the treatment? Why / why not?
      - The efficacy of the treatment? Why / why not?
    - **If patient initiated discussion about the treatment**: I noticed that you discussed [**INSERT LTP TREATMENT**] during the consultation when the patient brought it up
      - What did you think about the patient bringing up this treatment as part of the conversation? Why?
      - How did you feel about that patient bringing up this treatment as part of the conversation? Why?
      - If the patient hadn’t brought up this treatment, would you have brought it up as part of the discussion during this consultation? Why / why not?

**

**Moderator: repeat questions between * and ** for each LTP treatment discussed**

- **PLACEHOLDER FOR ANY QUESTIONS IDENTIFIED WHILST VIEWING CONSULTATION**
- **If HCP only discussed 1 LTP treatment**: Why did you only discuss [INSERT TREATMENT] with this patient? Why didn’t you discuss any other LTP options with this patient?

Let’s now discuss the consultation that you had with PATIENT 2.

**Moderator, repeat above questions for patient 2.**

**Ask patient 2 (Stephanie Huber) if relevant based on the consultation had:**

- If she wasn’t travelling as often for work, how would that impact whether LTP was recommended and which LTP would be recommended?
- **For interview 2 (before we changed PRF to 4 attacks):** If she was experiencing only 4 attacks, how would that impact whether LTP was recommended and which LTP would be recommended?
- If she was thinking of having children within the next year, how would that impact whether LTP was recommended and which LTP would be recommended?

**All:**

- **If HCP says that <6 attacks isn’t indicated, ask**: what is that belief based on / where has that information come from?

Section 4: Current practice (10 minutes)

***Objective: to understand use of LTP in practice, including when LTP is considered, and the treatments discussed***

In the final part of the interview, I’d like to understand a bit more about the conversations you have in your current practice with HAE patients, specifically with regard to LTP.

In your current practice, who typically initiates conversations about LTP?

- Patient/caregiver? Doctor?

What are the main factors that determine whether or not you offer LTP to a given patient?
**Allow for spontaneous response and then probe on:**

- Those with a certain attack frequency? What level? Why?
- Those with a certain attack location / severity? What location / severity? Why?
- Impact of the HAE attacks on patient QoL? What impact? Why? Anything else?

Which patients do you offer LTP to? Why?

Which patients don’t you offer LTP to? Why?

Which LTP treatments do you discuss with your patients?

How do you decide which specific LTP treatments to discuss with your patients?
**Allow for spontaneous response, and then show showcard 1.**

- Please can you read the following factors and rank them from 1-7 in terms of the extent to which you take these into account when deciding what LTP treatments to discuss with your patients? 1 = the most considered and 7 = the least considered.
  **Moderator, complete showcard 1**

**For each treatment mentioned:**

- What information do you discuss with patients about this treatment?
- What are your expectations of this treatment?
  - What do you communicate to your patients about your expectations of this treatment?
  - To what extent, if at all, does this treatment meet your expectations in practice? Why do you say that?

Thinking about the discussions that you have with patients about LTP, what, if any, are the challenges / difficulties that you face when having these conversations? Why do you say that?

- How, if at all, could those challenges be addressed?

Once you have initiated a patient on LTP, how often do you have follow-up appointments with the patient?

- How, if at all, does this differ depending on the LTP treatment the patient is taking? Why?

What do you typically discuss with patients during these follow up appointments? Why?

- How, if at all, does this differ depending on the LTP treatment the patient is taking? Why?

Finally, I’d like to understand whether you’re aware of any recent guideline changes relating to the use of LTP for HAE management.

- **If yes:** what guideline changes are you aware of / what did they say?
  - How, if at all, has that impacted your treatment approach with HAE patients? Why?
- **If no or if changes relating to recommended 1^st^ line/2^nd^ line LTPs not referred to, or recommendation that patients are evaluated for LTP at every visit not referred to, say:** the WAO/EAACI updated its guidelines in January 2022 relating to the use of LTP to say the following:
  **Moderator, show SHOWCARD 2
  Moderator, say:** acute treatment guidelines were also updated recently, but I would like to focus on the changes made to LTP guidelines.
  - Is this change to LTP guidelines something that you were aware of?
    - **If yes:** how, if at all, has this impacted your treatment approach with HAE patients? Why?
    - **If no:** how, if at all, do you think this change will impact your treatment approach with HAE patients in the future? Why do you say that?

**Thank you for taking part in this interview. I am now able to confirm that the pharmaceutical company sponsoring this research is BioCryst Pharmaceuticals, Inc.**

***THANK AND CLOSE INTERVIEW (CONTINUE RECORDING)***

Following completion of the interview with the HCP, please state on the audio file the respondent number and the number and type of adverse events you have noted and intend to report.

Then please ensure you report these adverse events according to the reporting procedure in which you have received training.

Following completion of an interview in which you believe there to have been NO adverse events, please state clearly on the audio file that you are not reporting any adverse events.

END AUDIO RECORDING

**SHOWCARD 1**

|  | **Factors considered** | Ranking (1-7) |
| --- | --- | --- |
| 1 | Reduction in attack frequency |  |
| 2 | Improvement in patient quality of life |  |
| 3 | Burden of treatment on patient quality of life |  |
| 4 | Side effects / tolerability |  |
| 5 | Long-term safety |  |
| 6 | Mode of administration |  |
| 7 | Frequency of administration |  |

**SHOWCARD 2**

We recommend –

- That the goals of treatment are to achieve total control of the disease and to normalize patients’ lives
- That patients are evaluated for long‐term prophylaxis at every visit, taking disease activity, burden, and control as well as patient preference into consideration
- The use of the following as first-line long-term prophylaxis (LTP)
  - Plasma-derived C1 inhibitors
  - Lanadelumab
  - Berotralstat
- The use of androgens only as second‐line long‐term prophylaxis
- All patients who are using LTP be routinely monitored for disease activity, impact, and control to inform optimization of treatment dosages and outcomes
