# Supplementary material for: Optimizing conversations on treatment management in hereditary angioedema: healthcare professional and patient perspectives on long-term prophylaxis and shared decision-making
Source: Allergy Asthma Clin Immunol. 2026 Jul 24;22:44. doi: 10.1186/s13223-026-01049-7 (PMC13401321; doi:10.1186/s13223-026-01049-7)
Supplement: Supplementary file 2 — Supplementary Material 2 [file 13223_2026_1049_MOESM2_ESM.docx]

**Additional File 3.** Patient discussion guide

Introduction (2 minutes)

***Objective: introduction, AE reporting & compliance***

START RECORDING

Good morning / afternoon, thank you for agreeing to take part in this interview. Before we begin, I would like to remind you of a few things that will have already been communicated to you prior to today.

We are conducting this research on behalf of a pharmaceutical company in order to understand more about the conversations that you have with healthcare professionals about hereditary angioedema (HAE). The interview will last approximately 30 minutes, and you will receive an incentive as a token of appreciation for your time and contribution to the project.

This interview is for market research purposes only – it is in no way intended to be promotional.

There are no right or wrong answers, we are just interested in your thoughts and opinions, and anything you do say will be kept confidential. You have the right to withhold information as you see fit or to withdraw from the interview at any time.

We will be audio recording the interview today, and [FOR PILOT INTERVIEWS: there are some interested colleagues and clients listening into this interview]; however, please be assured that your identity will remain anonymous.

We would prefer not to reveal the name of the sponsoring pharmaceutical company until the end of the interview, just in case knowing this biases any responses.

**Adverse events**

We are required to pass on to the sponsoring client any details of side effects or product complaints relating to their products that are mentioned during the interview. This is to help them learn more about the safety of their medicines. If this happens, we will need to collect details and report the side effects or product complaint.

Section 1 – patient background (5 minutes)

***Objective: understand how long the patient has been diagnosed with HAE, their treatment history, and use of acute treatment***

**Ask respondent(s) to introduce self anonymously (i.e. using first name only):**

Bearing in mind that this is an anonymous interview:

**Ask patients:**

- Please can you briefly tell me a bit about yourself
  - Age?
  - Whether studying or at work?
  - Who do you live with at home?
  - Hobbies / interests?
- I understand that you’ve been diagnosed by a healthcare professional as having hereditary angioedema (HAE) – how old were you when you were diagnosed with HAE?
- How many HAE attacks have you had in the last month?
- Do you know what specialty your HAE doctor is?
- Do you know how many HAE patients your HAE doctor treats?

**Note to moderator, all patients have been screened to ensure that they have been prescribed acute treatment. Note down acute treatment currently used.**

How often do you take your acute treatment?

- Do you treat **all** HAE attacks that you have, with acute treatment? Why / why not?
  - **If no:** how do you decide which attacks to treat with acute treatment, and which ones not to
- How bad do your symptoms need to get before you decide to take your acute treatment e.g. do you take treatment as soon as initial symptoms appear, or do you wait until symptoms have developed into a full attack? Why?

**Moderator, refer to screening data and say:**

- **If respondent hasn’t ever received long-term prophylaxis (LTP):** I understand that you have discussed LTP with your HAE doctor, but that you have never taken long term prophylactic treatment – is that correct?
- **If respondent has received LTP in the past, but not currently**: I understand that you took LTP that was prescribed by your HAE doctor in the past, to help prevent HAE attacks from occurring - is that correct?
  - Which LTP(s) were you prescribed?
    **Moderator, establish LTPs taken and the order in which they were taken**
- **If respondent currently receives LTP**: I understand that you currently take LTP treatment that has been prescribed by your HAE doctor to help prevent HAE attacks from occurring, is that correct?
  - Which LTP do you take?
  - Have you taken any other LTP treatments in the past? Which ones?

**Moderator, establish LTPs taken and the order in which they were taken**

**Moderator, a mix of LTP-experienced patients have been recruited (never taken [but have discussed], took in the past, currently taking) – if the respondent’s answers to the above questions are not correct, please re-classify the respondent and inform RP. Terminate if respondent has never taken AND never discussed LTP (this shouldn’t be the case as respondents are being screened to ensure that they have at least discussed LTP with their HAE doctor.)**

Section 2 – conversations had about LTP (18 minutes)

***Objective: to understand the conversations had with their HAE doctor about LTP, what was discussed during each conversation, how the patient felt about it, and why they did / didn’t start taking it***

I would like to understand more about the conversations that you’ve had with your HAE doctor about LTP specifically.

How long ago was LTP first discussed with your HAE doctor?
*

- Who raised the topic of LTP at this time? You / the doctor?

**If the patient raised the topic:**

- - What did you say? Why did you say that?
  - How did your HAE doctor respond? What did they say?
  - How did that make you feel? Why?
  - What questions or concerns did you have in response?
  - To what extent, if at all, did the doctor answer your questions / concerns? What did they say?
  - What information / support were you provided with at this time?
  - Overall, how were you left feeling after this discussion? Why?

**If the HAE doctor raised the topic:**

- - What did your HAE doctor say?
  - How did that make you feel? Why?
  - What questions / concerns did you have in response? What did you say?
  - To what extent, if at all, did the doctor answer your questions / concerns? What did they say?
  - What information / support were you provided with at this time?
  - Overall, how were you left feeling after this discussion? Why?

**If not already discussed:**

- Did your HAE doctor discuss specific LTP treatments with you at this time?
  - **If yes:** which ones?

**For each treatment discussed, ask:**

- - - What did your HAE doctor discuss with you about this specific treatment?

**Moderator, ensure you understand if the patient had to make a decision between LTP treatments and how they came to that conclusion e.g. how did the doctor present the options**

**

**If patient has never received LTP:**

- Why didn’t you start taking LTP at that time?
- What, if anything, could have helped you feel more comfortable with the idea of taking LTP treatment at this time e.g. further information / support? Why?
- Have you discussed LTP with your HAE doctor since that initial conversation?
  - **If yes, repeat questions between * and ** and then ask:**
    - How long ago did this conversation happen?
    - Why didn’t you start taking LTP at that time?
    - What, if anything, could have helped you feel more comfortable with the idea of taking LTP treatment at this time e.g. further information / support? Why?
    - Have you discussed LTP with your HAE doctor since this conversation?

**If yes, repeat above questioning until all LTP discussions understood**

*continued below*

- What would need to happen, or what information or support would you need to be provided with to help you feel more comfortable with the idea of taking LTP in the future? Why do you say that?
  - How many attacks would you have to have within a three-month period to consider taking LTP treatment? Why do you say that?

**If patient has received LTP ask:** did you start taking LTP at that time? Why / Why not?

***

- **If yes:**
  - What LTP did you start taking?
  - What encouraged you to start taking that treatment? Why?
  - Was there anything specific that the HCP said, did, or showed you that encouraged you to start taking this treatment? What? Why?
  - What were your expectations of this treatment at that time e.g. what were you hoping that it would achieve? Why?
    - To what extent, if at all, were those expectations met? Why do you say that?
  - To what extent, if at all, did your HAE doctor discuss their expectations of the treatment with you? What did they say?
    - To what extent, if at all, did your HAE doctor discuss any potential side effects with this treatment and how to address them? What did they say?
  - How often did you have follow-up appointments with your HAE doctor, or another healthcare professional? Which healthcare professional?
- **If no:**
  - Why didn’t you start taking LTP at that time?
  - What, if anything, could have helped you feel more comfortable with the idea of taking LTP treatment at this time e.g. further information / support? Why?

****

- When did you next discuss LTP with your HAE doctor?

**Repeat questions between * and ** and then ask:**

- - Did you start taking LTP at that time? Why / why not?
  - **Repeat questions between *** and ******

**Repeat above questioning until all LTP discussions understood.**

**For those who took LTP in the past, but not currently, we need to understand conversations had about LTP all the way up to stopping LTP.**

**For those who currently take LTP we need to understand conversations had about LTP all the way up to the current day.**

**If patient has received LTP in the past, but not currently:**

- Why did you stop taking LTP treatment?
- To what extent was this your decision vs. the HAE doctor’s decision?
- Have you discussed LTP treatment with your HAE doctor since stopping LTP?
  - **If yes:**
    - What did you discuss?
    - How did that make you feel? Why?
- To what extent, if at all, would you consider taking LTP treatment again in the future? Why / why not?
  - What would need to happen, or what information or support would you need to be provided with, in order for you to feel more comfortable with the idea of taking LTP in the future? Why do you say that?
  - How many attacks would you have to have within a three-month period to consider taking LTP treatment? Why do you say that?

Section 3 – ‘In their shoes’ projective exercise (5 minutes)

***Objective: understand hypothetical reasons as to why patients may accept / reject LTP recommendations***

**Say to those currently on LTP:**

I would like you to imagine that you’re talking to a HAE patient similar to you. This person has been recommended LTP by their HAE doctor but is reluctant to take it.

How would you explain the pros and cons of LTP treatment to this patient, based on your experience?

**Allow for spontaneous response and then probe on:**

- What, if any, are the biggest benefits of LTP in your experience?
- What, if any, are the biggest drawbacks of LTP in your experience?

Finally, I would like you to step into the shoes of n HAE patient who is similar to you in every respect, other than the fact that they don’t currently receive LTP to manage their HAE.

- Why do you think this patient wouldn’t take LTP? What would their reservations be? Why?
- What, if any, information or support would they be lacking from their HAE doctor?
- What, if any, other information or support might they be lacking?
- What else could help this patient feel more comfortable with the idea of taking LTP treatment? Why do you say that?

**Say to those not currently on LTP:**

In the final part of the interview, I would like you to step into the shoes of an HAE patient who is similar to you in every respect, other than the fact that they currently receive LTP to manage their HAE.

- Why do you think this patient would take LTP? What would have helped this patient feel more comfortable with the idea of taking LTP? Why do you say that?
- What, if any, information or support would their HAE doctor have provided them with?
- What, if any, other information or support would they have been provided with?
- What else do you think might have helped this patient feel more comfortable with the idea of taking LTP treatment? Why do you say that?

**Additional questions asked in some pilot interviews:**

- **If patient satisfied:** what would make them consider a switch of LTP product?
- **If doctor has not spoken about new LTP options:** would it be desirable for your doctor to update you on new LTP treatments?

**Thank you for taking part in this interview. I am now able to confirm that the pharmaceutical company sponsoring this research is BioCryst Pharmaceuticals, Inc.**

***THANK AND CLOSE INTERVIEW (CONTINUE RECORDING)***

Following completion of the interview with the respondent please state on the audio file the respondent number and the number and type of adverse events you have noted and intend to report.

Then please ensure you report these adverse events according to the reporting procedure in which you have received training.

Following completion of an interview in which you believe there to have been NO adverse events, please state clearly on the audio file that you are not reporting any adverse events.

END AUDIO RECORDING
