# Supplementary material for: Optimizing conversations on treatment management in hereditary angioedema: healthcare professional and patient perspectives on long-term prophylaxis and shared decision-making
Source: Allergy Asthma Clin Immunol. 2026 Jul 24;22:44. doi: 10.1186/s13223-026-01049-7 (PMC13401321; doi:10.1186/s13223-026-01049-7)
Supplement: Supplementary file 3 — Supplementary Material 3 [file 13223_2026_1049_MOESM3_ESM.docx]

**Additional File 1** Patient personas
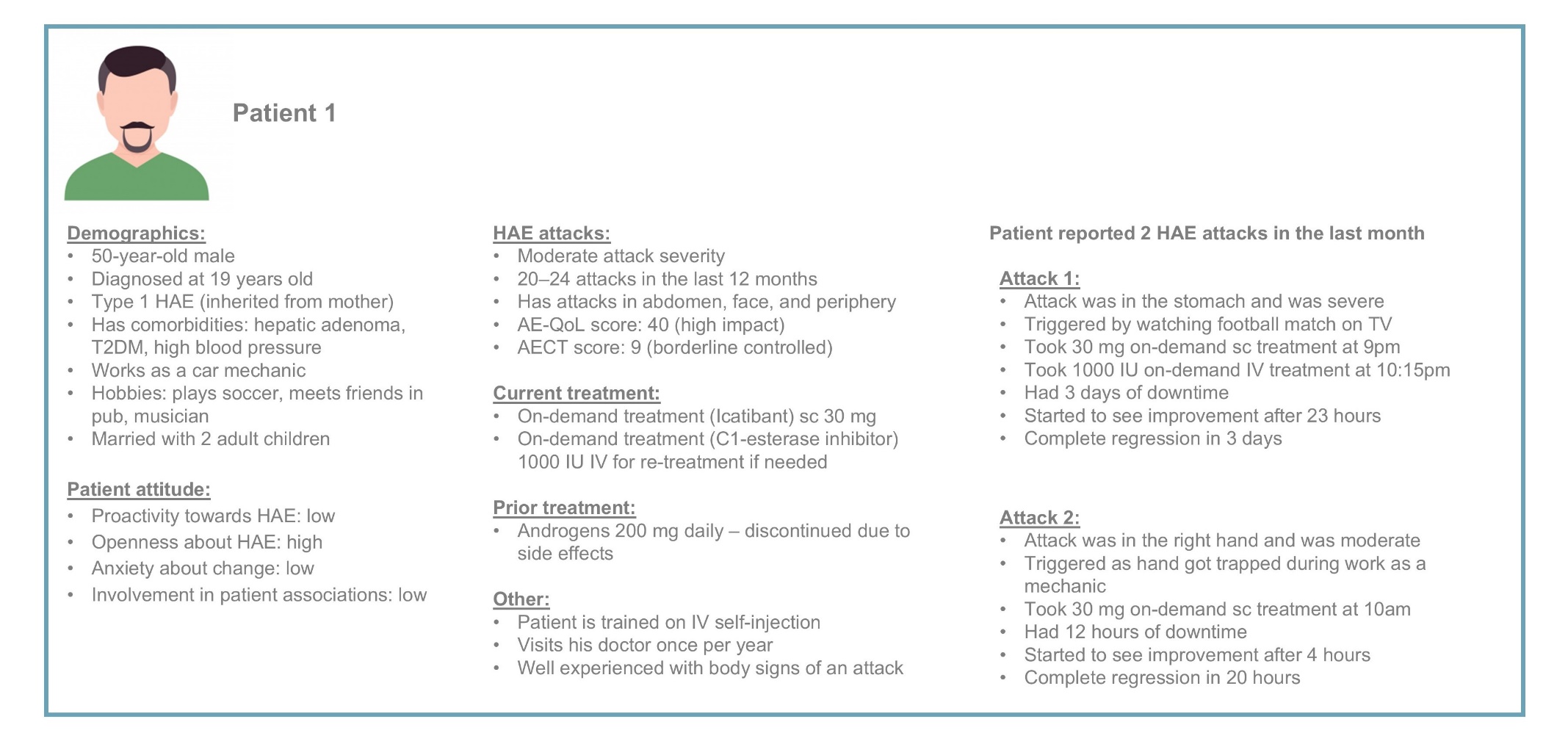

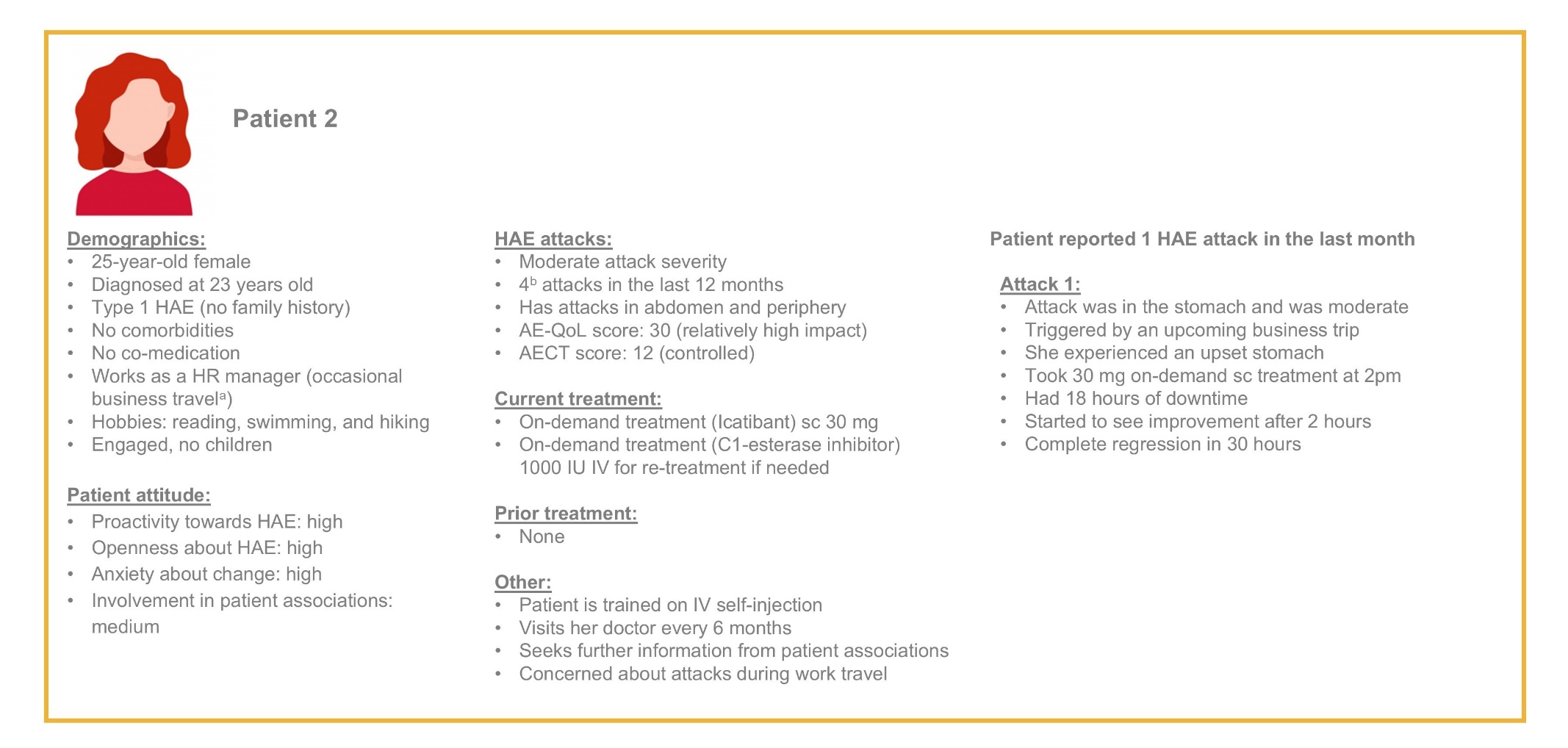
AECT, Angioedema Control Test; AE-QoL, Angioedema quality of life; HAE, hereditary angioedema; HR, human resources; IV, intravenous; sc, subcutaneous; T2DM, type 2 diabetes mellitus

^a^In the first three interviews, the patient presented with ‘regular business travel’

^b^In the first two interviews, the patient presented with 6 attacks in the last 12 months
